# Curated eutherian third party data gene data sets

**DOI:** 10.1016/j.dib.2015.11.056

**Published:** 2015-12-11

**Authors:** Marko Premzl

**Affiliations:** Laboratory of Genomics, Centre of Animal Reproduction, 55 Heinzel St., Zagreb, Croatia

**Keywords:** Comparative Genomic Analysis, Gene Annotations, Molecular Evolution, Phylogenetic Analysis

## Abstract

The free available eutherian genomic sequence data sets advanced scientific field of genomics. Of note, future revisions of gene data sets were expected, due to incompleteness of public eutherian genomic sequence assemblies and potential genomic sequence errors. The eutherian comparative genomic analysis protocol was proposed as guidance in protection against potential genomic sequence errors in public eutherian genomic sequences. The protocol was applicable in updates of 7 major eutherian gene data sets, including 812 complete coding sequences deposited in European Nucleotide Archive as curated third party data gene data sets.

**Specifications Table**

**Table:** 

Subject area	Biology
More specific subject area	Genomics
Type of data	Third party data
How data was acquired	*In computo*
Data format	FAS, TXT
Experimental factors	Eutherian comparative genomic analysis protocol
Experimental features	Curated gene data sets
Data source location	N/A
Data accessibility	The original gene data sets were deposited in European Nucleotide Archive under accession numbers: FR734011-FR734074 (http://www.ebi.ac.uk/ena/data/view/FR734011-FR734074), HF564658-HF564785 (http://www.ebi.ac.uk/ena/data/view/HF564658-HF564785), HF564786-HF564815 (http://www.ebi.ac.uk/ena/data/view/HF564786-HF564815), HG328835-HG329089 (http://www.ebi.ac.uk/ena/data/view/HG328835-HG329089), HG426065-HG426183 (http://www.ebi.ac.uk/ena/data/view/HG426065-HG426183), HG931734-HG931849 (http://www.ebi.ac.uk/ena/data/view/HG931734-HG931849) and LM644135-LM644234 (http://www.ebi.ac.uk/ena/data/view/LM644135-LM644234). Data analysis is with this article.

**Value of the data**

• Curated gene data sets applicable in gene annotations and genome analyses.
• Curated gene data sets applicable in phylogenetic analyses.
• Curated gene data sets applicable in protein structure and function analyses.

## 1 Data

**Fig. 1:**
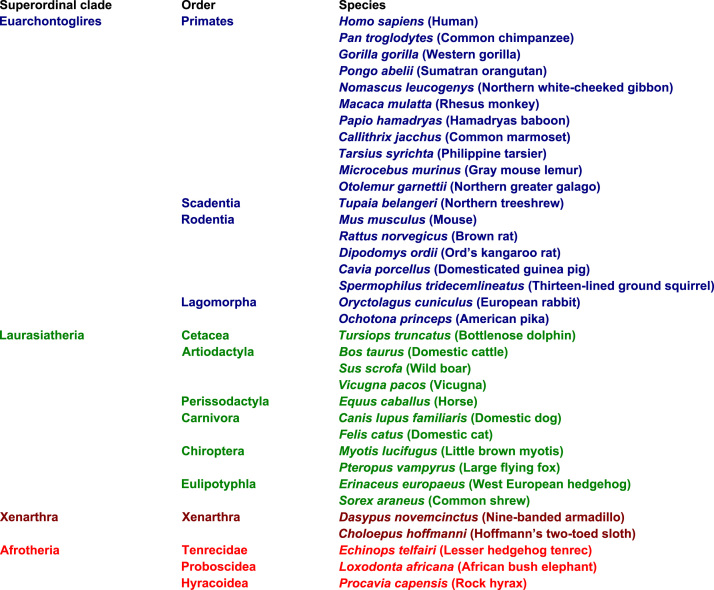
Public eutherian genomic sequence assemblies (http://www.ensembl.org).

**Fig. 2:**
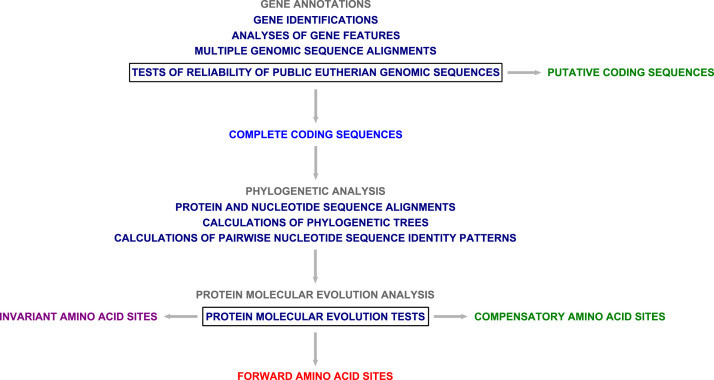
Eutherian comparative genomic analysis protocol scheme.

**Fig. 3:**
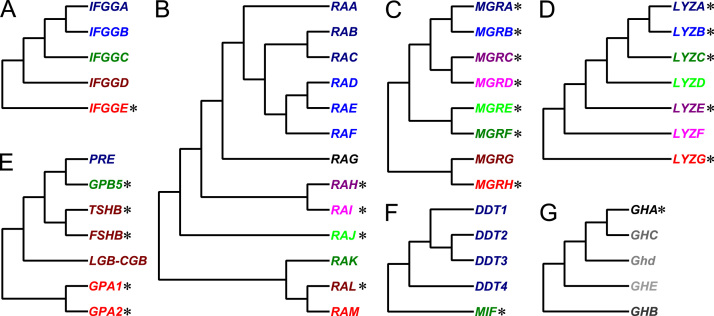
Revised gene classifications of eutherian interferon-γ-inducible GTPase genes (A), ribonuclease A genes (B), Mas-related G protein-coupled receptor genes (C), lysozyme genes (D), adenohypophysis cystine-knot genes (E) and growth hormone genes (G) and human D-dopachrome tautomerase and macrophage migration inhibitory factor genes (F). The major gene clusters with no evidence of differential gene expansions were indicated by *s.

Undoubtedly, the eutherian comparative genomics momentum was maintained by programmatic, considerable international efforts in production, assembly and analysis of public eutherian genomic sequence data sets (Fig. 1) [1], [2], [3]. For example, the initial sequencing and analysis of human genome revised human gene data sets [4], [5]. Nevertheless, these analyses were subject to future updates and revisions due to incompleteness of public eutherian genomic sequence data sets and potential genomic sequence errors [1], [2], [3], [4], [5], [6]. The eutherian comparative genomic analysis protocol was proposed as guidance in protection against potential genomic sequence errors in public eutherian genomic sequences [7], [8], [9], [10], [11], [12]. The protocol was established as one framework of eutherian third party data gene data set descriptions (Fig. 2). The protocol included new genomics and protein molecular evolution tests applicable in updates and revisions of 7 major eutherian gene data sets, including interferon-γ-inducible GTPase genes, ribonuclease A genes, Mas-related G protein-coupled receptor genes, lysozyme genes, adenohypophysis cystine-knot genes, macrophage migration inhibitory factor and D-dopachrome tautomerase genes and, finally, growth hormone genes (Fig. 3). The protocol discriminated major gene clusters with and without evidence of differential gene expansions. For example, the eutherian major gene clusters with no evidence of differential gene expansions could be suitable in phylogenomic analyses.

## 2 Experimental design, materials and methods

The eutherian comparative genomic analysis protocol included gene annotations, phylogenetic analysis and protein molecular evolution analysis [7], [8], [9], [10], [11], [12] (Fig. 2). The protocol used free available eutherian genomic sequence data sets deposited in public biological databases and software.

## 3 Gene annotations

The gene annotations included gene identifications in eutherian genomic sequences, analyses of gene features, tests of reliability of eutherian public genomic sequences and multiple pairwise genomic sequence alignments. The BioEdit program was used in nucleotide and protein sequence analyses (http://www.mbio.ncsu.edu/BioEdit/bioedit.html). The NCBI׳s BLAST programs were used in identifications of genes in eutherian genomic sequence assemblies downloaded from NCBI (ftp://ftp.ncbi.nlm.nih.gov/blast/ and ftp://ftp.ncbi.nlm.nih.gov/genbank/genomes/Eukaryotes/vertebrates_mammals/). In addition, the Ensembl genome browser׳s BLAST or BLAT programs were used in gene identifications (http://www.ensembl.org). The analyses of gene features included direct evidence of eutherian gene annotations deposited in NCBI׳s nr, est_human, est_mouse and est_others databases (http://www.ncbi.nlm.nih.gov). The new tests of reliability of eutherian public genomic sequences tested potential coding sequences using genomic sequence redundancies. First, the tests analysed nucleotide sequence coverage of potential coding sequences using primary experimental sequence reads deposited in NCBI׳s Trace Archive (http://www.ncbi.nlm.nih.gov/Traces/trace.cgi) and BLAST programs. Second, the potential coding sequences were classified as complete coding sequences only if consensus trace sequence coverage was available for every nucleotide. Alternatively, the potential coding sequences were described as putative coding sequences. Only the complete coding sequences were deposited in European Nucleotide Archive as curated third party data gene data sets (http://www.ebi.ac.uk/ena/about/tpa-policy) and used in phylogenetic and protein molecular evolution analyses. In revised eutherian gene nomenclatures, the guidelines of human and mouse gene nomenclature were used (http://www.genenames.org/about/guidelines and http://www.informatics.jax.org/mgihome/nomen/gene.shtml). The maskings of transposable elements using RepeatMasker program were included as preparatory steps in multiple pairwise genomic sequence alignments (http://www.repeatmasker.org/). The RepeatMasker׳s default settings were used, except simple repeats and low complexity elements were not masked. The mVISTA program was used in genomic sequence alignments, using AVID alignment algorithm and default settings (http://genome.lbl.gov/vista/index.shtml). Using ClustalW implemented in BioEdit, the common predicted promoter genomic sequence regions were aligned at nucleotide sequence level and then manually corrected. The pairwise nucleotide sequence identities of common predicted promoter genomic sequence regions calculated using BioEdit were used in statistical analyses (Microsoft Office Excel).

## 4 Phylogenetic analysis

The phylogenetic analyses included protein and nucleotide sequence alignments, calculations of phylogenetic trees and calculations of pairwise nucleotide sequence identity patterns. First, the translated complete coding sequences were aligned at amino acid level using ClustalW implemented in BioEdit. The protein sequence alignments were manually corrected, as well as nucleotide sequence alignments. The MEGA program was used in phylogenetic tree calculations (http://www.megasoftware.net), using neighbour-joining method (default settings, except gaps/missing data treatment=pairwise deletion), minimum evolution method (default settings, except gaps/missing data treatment=pairwise deletion) and maximum parsimony method (default settings, except gaps/missing data treatment=use all sites). The pairwise nucleotide sequence identities of complete coding sequences were calculated using BioEdit and used in statistical analysis (Microsoft Office Excel).

## 5 Protein molecular evolution analysis

The protocol included new protein molecular evolution tests integrating patterns of nucleotide sequence similarities with protein tertiary structures. The MEGA program was used in calculations of codon usage statistics. Specifically, the ratios between observed and expected amino acid codon counts determined relative synonymous codon usage statistics (*R*) that indicated amino acid codons with *R≤*0.7 as not preferable amino acid codons. In reference protein amino acid sequences, there were invariant amino acid sites (invariant alignment positions), forward amino acid sites (variant alignment positions that did not include not preferable amino acid codons) and compensatory amino acid sites (variant alignment positions that included not preferable amino acid codons). The presence of preferable amino acid codons, as well as absence of not preferable amino acid codons indicated that forward amino acid sites could have major influence on protein tertiary structures and functions. The DeepView/Swiss-PdbViever was used in analyses of protein tertiary structures (http://spdbv.vital-it.ch/).
