# Supplementary material for: Curated eutherian third party data gene data sets
Source: Data Brief. 2015 Dec 11;6:208–13. doi: 10.1016/j.dib.2015.11.056 (PMC4707174; doi:10.1016/j.dib.2015.11.056)
Supplement: Supplementary file 1 — Supplementary material [file mmc1.zip › 20151118_Conflict_of_Interest_edited_manuscript_V2.pdf]

Conflict of Interest

No conflict of interest was declared.
